# Supplementary material for: Reducing voltage-dependent potassium channel Kv3.4 levels ameliorates synapse loss in a mouse model of Alzheimer’s disease
Source: Brain Neurosci Adv. 2022 Mar 24;6:23982128221086464. doi: 10.1177/23982128221086464 (PMC8961358; doi:10.1177/23982128221086464)
Supplement: sj-pdf-1-bna-10.1177_23982128221086464 – Supplemental material for Reducing voltage-dependent potassium channel Kv3.4 levels ameliorates synapse loss in a mouse model of Alzheimer’s disease [file sj-pdf-1-bna-10.1177_23982128221086464.pdf]

**Supplementary table 1:** Human brain samples used for western blot analysis

| | BBN | Sex | Age | PMI (hours) | Braak Stage |
|---|---|---|---|---|---|
| Braak 0 - 1 | 001.35138 | F | 73 | 74 | 0 |
| | 001.34150 | M | 63 | 115 | 0 |
| | 001.35420 | F | 82 | 114 | I |
| | 001.35215 | M | 82 | 40 | I |
| | 001.31504 | F | 65 | 76 | I |
| | 001.31503 | F | 57 | 73 | I |
| | 001.28402 | M | 79 | 49 | I |
| | 001.28794 | F | 79 | 61 | I |
| | 001.30208 | M | 73 | 66 | I |
| | 001.30178 | M | 72 | 60 | 0 |
| Braak 3 - 4 | 001.32820 | F | 69 | 94 | IV |
| | 001.29528 | F | 93 | 31 | III |
| | 001.28411 | F | 88 | 65 | III |
| | 001.28405 | M | 84 | 83 | IV |
| | 001.26499 | F | 81 | 59 | III |
| | 001.26491 | M | 77 | 27 | III |
| | BBN_26492 | F | 74 | 71 | IV |
| | BBN_24323 | M | 67 | 103 | IV |
| | BBN_20994 | M | 75 | 59 | III |
| | BBN_19600 | F | 85 | 36 | III |
| Braak 5 - 6 | 001.35183 | M | 74 | 75 | VI |
| | 001.35096 | M | 72 | 103 | VI |
| | 001.33698 | F | 90 | 76 | VI |
| | 001.33636 | M | 93 | 43 | VI |
| | 001.32929 | F | 85 | 80 | VI |
| | 001.30883 | F | 61 | 69 | VI |
| | 001.29911 | M | 66 | 52 | VI |
| | 001.29521 | M | 95 | 96 | VI |
| | 001.26500 | M | 81 | 83 | VI |
| | BBN_24668 | F | 96 | 61 | VI |

**Supplementary Table 2: Sequences of qPCR primer pairs**

| PRIMER | DIRECTION | SEQUENCE |
|---|---|---|
| **KV3.4a** | Forward<br>Reverse | TCCACCACTCGAGACAGAAACAAG<br>ATGTGGCAGGAACAGAGTCC |
| **GAPDH** | Forward<br>Reverse | CCAAGGTCATCCATGACAAC<br>ACAGTCTTCTGGGTGGCAGT |
| **RPLP1** | Forward<br>Reverse | AGCCGGTGTAAATGTTGAGC<br>CAGATGAGGCTCCCAATGTT |

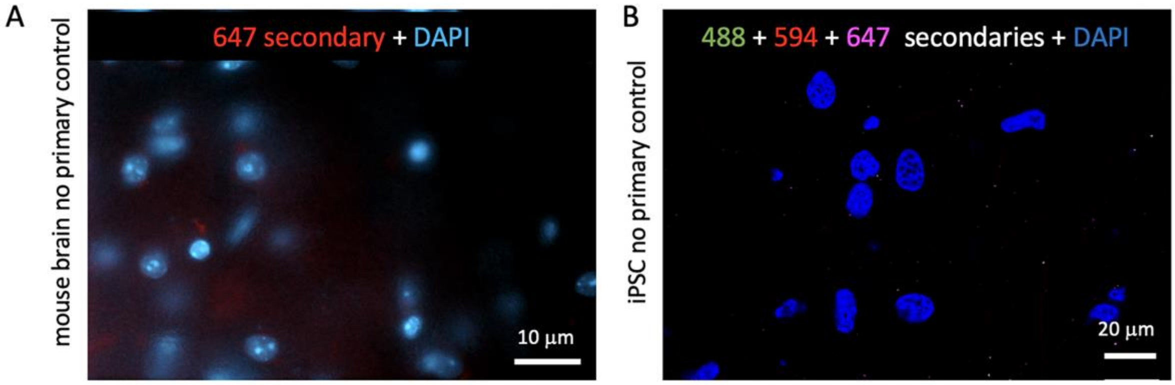


**Supplemental Figure 1:** No primary antibody negative controls for immunohistochemistry of mouse bran (A) and immunocytochemistry of iPSC derived neurons (B).

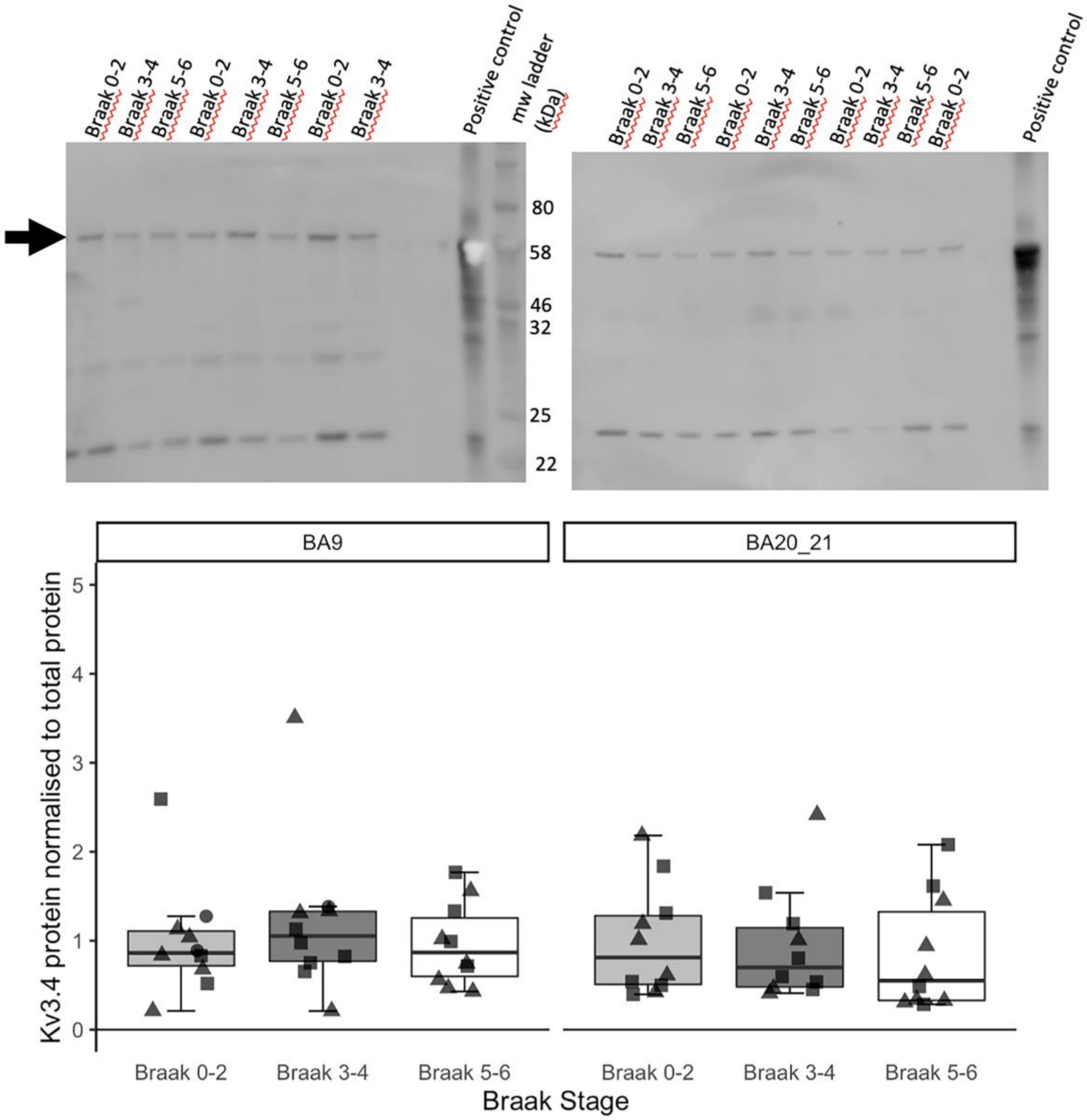


**Supplemental Figure 2:** Western blot of human brain samples from frontal (BA9) and temporal (BA20/21) cortex of people with low (Braak 0-2), moderate (Braak 3-4) and extensive (Braak 5-6) Alzheimer's disease pathology reveals no difference between brain regions or Braak stages (n=10 per group, linear model on Tukey transformed data $p>0.05$ for all fixed effects). The arrow indicates full-length $K_V3.4$ protein immunoreactivity.
